# The Effects of Vortioxetine on Cognitive Function in Patients with Major Depressive Disorder: A Meta-Analysis of Three Randomized Controlled Trials

**DOI:** 10.1093/ijnp/pyw055

**Published:** 2016-06-15

**Authors:** RS McIntyre, J Harrison, H Loft, W Jacobson, CK Olsen

**Affiliations:** University Health Network, University of Toronto, Canada (Dr McIntyre);; Metis Cognition Ltd., Kilmington Common, UK & Alzheimer Center, VU University Medical Center, Amsterdam, the Netherlands (Dr Harrison); H Lundbeck A/S, Copenhagen, Denmark (Drs Loft and Olsen); Takeda Development Center Americas, Deerfield, IL (Dr Jacobson).

**Keywords:** cognitive function, duloxetine, major depressive disorder, meta-analysis, vortioxetine

## Abstract

**Background:**

Management of cognitive deficits in Major Depressive Disorder (MDD) remains an important unmet need. This meta-analysis evaluated the effects of vortioxetine on cognition in patients with MDD.

**Methods:**

Random effects meta-analysis was applied to three randomized, double-blind, placebo-controlled 8-week trials of vortioxetine (5–20mg/day) in MDD, and separately to two duloxetine-referenced trials. The primary outcome measure was change in Digit Symbol Substitution Test (DSST) score. Standardized effect sizes (SES) versus placebo (Cohen’s *d*) were used as input. Path analysis was employed to determine the extent to which changes in DSST were mediated independently of a change in Montgomery-Åsberg Depression Rating Scale (MADRS) score. Meta-analysis was applied to MADRS-adjusted and -unadjusted SES values. Changes on additional cognitive tests were evaluated (source studies only).

**Results:**

Before adjustment for MADRS, vortioxetine separated from placebo on DSST score (SES 0.25–0.48; nominal *p* < 0.05) in all individual trials, and statistically improved DSST performance versus placebo in meta-analyses of the three trials (SES = 0.35; *p* < 0.0001) and two duloxetine-referenced trials (SES = 0.26; *p* = 0.001). After adjustment for MADRS, vortioxetine maintained DSST improvement in one individual trial (*p* = 0.001) and separation from placebo was maintained in meta-analyses of all three trials (SES = 0.24; *p* < 0.0001) and both duloxetine-referenced trials (SES 0.19; *p* = 0.01). Change in DSST with duloxetine failed to separate from placebo in individual trials and both meta-analyses. Change in DSST statistically favored vortioxetine versus duloxetine after MADRS adjustment (SES = 0.16; *p* = 0.04).

**Conclusions:**

Vortioxetine, but not duloxetine, significantly improved cognition, independent of depressive symptoms. Vortioxetine represents an important treatment for MDD-related cognitive dysfunction.

## Introduction

Individuals with Major Depressive Disorder (MDD) often exhibit impairments in cognitive function, including executive function, processing speed, concentration/attention, learning, and memory (National Academies of Sciences, Engineering, and Medicine, 2015). The foregoing deficits are key contributors to social, functional, and occupational disability seen in many affected individuals. Cognitive impairment may serve as a “mediational nexus” between MDD and poor functional outcomes—particularly occupational and relational difficulties—even when depressive symptoms have subsided (Boartolato et al., 2014). In particular, deficiencies in memory and the cognitive substrates of information processing and verbal fluency reduce the capacity for normal daily functioning (National Academies of Sciences, Engineering, and Medicine, 2015), while the impact on family and social interactions can strain relationships and lead to household tension (Hammar and Ardal, 2009; Lam et al., 2014). Outside the home, cognitive deficits impair workplace performance, with implications for reduced productivity and an elevated burden of absenteeism (McIntyre et al., 2013; National Academies of Sciences, Engineering, and Medicine, 2015). Indeed, among working adults with MDD, measures of cognitive dysfunction may be a greater determinant of presenteeism/absenteeism than is the total depression severity score (McIntyre et al., 2015b). More broadly, epidemiological evidence supports an association between recurrent MDD and dementing disorders (Alexopoulos et al., 1993; Sáez-Fonseca et al., 2007) through mechanisms believed to stem from corticosteroid neurotoxicity (Korczyn and Halperin, 2009), although it is not known whether cognitive disturbances in young adults with MDD identify a subpopulation at greater risk for later dementing disorders. In the sphere of public health, compromised executive function and attention, slowed information processing, and poor judgment have important safety implications, increasing the risk of motor vehicle accidents, falls, and medication errors (Tyson et al., 2012). Available evidence also indicates that health service utilization and costs related to healthcare expenditure are greater in individuals with MDD and cognitive dysfunction when compared to those who have MDD but no cognitive deficits (Xiang and An, 2015).

In light of the far-reaching impact of cognitive dysfunction in MDD, close and continued monitoring of cognitive function should be prioritized in the working population and the depressed elderly. Treatment guidelines also stress restoration of psychosocial functioning as an essential goal in maximizing functional recovery (American Psychiatric Association, 2000a; Patten et al., 2009). Importantly, the development of interventions that can improve cognitive function in individuals with MDD can be expected to improve psychosocial outcomes and, possibly, workplace functioning and public safety, as well as reducing health-related costs and expenditures.

Despite the large and expanding array of approved antidepressants, the availability of pharmacological agents that mitigate deficits in cognitive function remains an important unmet clinical need. The novel multimodal antidepressant vortioxetine has shown evidence of cognitive benefit in animal models, where it improved measures of memory (e.g. reversal learning; Wallace et al., 2014; Sanchez et al., 2015), as well as in patients with MDD (Katona et al., 2012; McIntyre et al., 2014; Mahableshwarkar et al., 2015). The effects of vortioxetine on cognitive dysfunction in patients with MDD are hypothesized to be mediated through its multimodal actions. *In vitro*, vortioxetine inhibits the serotonin (5-HT) transporter and acts as a serotonin receptor agonist (5-HT_1A_), a partial agonist (5-HT_1B_), and antagonist (5-HT_3_, 5-HT_7_ and 5-HT_1D_) at the indicated targets (Bang-Andersen et al., 2011). It is posited that the foregoing combination of pharmacologic actions and pharmacodynamic consequences underlies its antidepressant efficacy. The pharmacodynamic mechanisms proposed to mediate the effects of vortioxetine on cognitive dysfunction in patients with MDD are believed to include increased glutamate neurotransmission (via inhibition of gamma-aminobutyric acid [GABA] interneurons expressing 5-HT_3_ heteroreceptors) and neuroplasticity in brain regions relevant to cognitive function (e.g. hippocampus and prefrontal cortex; Haddjeri et al., 2012; Riga et al., 2013; Pehrson et al., 2015; Sanchez et al., 2015). Other mechanisms hypothesized to contribute to the pro-cognitive effects of vortioxetine in animal models include direct and/or indirect effects via serotonergic, noradrenergic, cholinergic, dopaminergic, and histaminergic systems (Sanchez et al., 2015).

The unique pharmacological profile of vortioxetine has led to conjecture that it may have beneficial effects on cognitive dysfunction in patients with MDD. Since such dysfunction is more pronounced in elderly patients with MDD than in younger depressed adults, this hypothesis was initially examined in a controlled, duloxetine-referenced trial in patients aged ≥65 years (Katona et al., 2012). In this trial, cognitive function was evaluated as a secondary endpoint (the primary endpoint assessed depressive symptoms) using two objective “pencil-and-paper” neuropsychological tests: the Digit Symbol Substitution Test (DSST), which requires the integrity of executive function, processing speed, attention, spatial perception, and visual scanning; and the Rey Auditory Verbal Learning Test (RAVLT), which evaluates short-term auditory-verbal memory and various aspects of learning and information retrieval. These tests were chosen based on evidence suggesting that duloxetine was superior to placebo on the RAVLT, but not on tests that have a greater impact on executive function (DSST, the Two-Digit Cancellation Test, and the Letter-Number Sequencing Test), including a symbol coding test equivalent to the DSST (Raskin et al., 2007). The trial in elderly patients showed that both vortioxetine and duloxetine improved depressive symptoms and had positive effects on the RAVLT, whereas only vortioxetine improved DSST performance. Based on these results, two large, well-controlled, similarly designed clinical trials that included a battery of objective cognitive tests and, in one case, an active reference (Mahableshwarkar et al., 2015), were initiated in adults <65 years old with MDD (McIntyre et al., 2014; Mahableshwarkar et al., 2015). Both demonstrated significant improvement in cognitive dysfunction, measured using the DSST (number of correct symbols) as either the primary endpoint (Mahbleshwarkar et al., 2015), or as part of a composite primary endpoint/multiplicity-controlled key secondary endpoint (McIntyre et al., 2014).

Evidence, although limited, suggests that mechanistically dissimilar antidepressants exert significantly positive effects on delayed recall and the cognitive substrates of psychomotor speed (Rosenblat et al., 2015). Domains of cognition can be independently evaluated in patients with MDD using objective and self-reported measures, which provide quantitative assessment of pre-treatment cognitive function as well as change in cognitive function with treatment. Whether an antidepressant exerts an independent and clinically relevant effect on cognitive function that is not mediated through improvement in other depressive symptoms has both conceptual and clinical implications, and several methodological aspects of clinical trial design are crucial in order to determine that an antidepressant has direct effects on cognitive dysfunction (McIntyre et al., 2015a). Trials should pre-specify cognition as the primary dependent measure, include a placebo arm and an active reference, and employ a statistical approach (e.g. path analysis) that adjusts for pseudo-specific effects that confound cognitive measures. To date, although clinical trials have been conducted to evaluate the effects of different antidepressants on cognitive function, not all have adjusted for other dimensions of MDD that may influence cognitive performance, e.g. the presence of psychiatric and/or medical comorbidity (McIntyre et al., 2013; Rosenblat et al., 2015).

The present meta-analysis was based on the results of three similarly designed, adequately powered, 8-week, randomized, double-blind, placebo-controlled trials of vortioxetine in patients with MDD (Katona et al., 2012; McIntyre et al., 2014; Mahableshwarkar et al., 2015). The three trials examined vortioxetine over a dose range of 5–20mg daily and used change on the DSST as a predefined outcome measure. DSST is an appropriate measure because it is impacted by several of the cognitive domains that are most profoundly impaired in MDD (i.e. executive function, processing speed, and attention) and can detect changes in cognitive function if treatment is effective, including in subsets of patients who are non-responders or non-remitters (McIntyre et al., 2013; Mahableshwarkar et al., 2015). These trials also evaluated a range of cognitive function measures other than the DSST, offering a more complete picture of the range of effects of vortioxetine on different domains of cognitive function in patients with MDD.

This meta-analysis evaluated the effects of vortioxetine on cognitive function in patients with MDD using statistical path analysis to control for concurrent change in depressive symptoms as measured by Montgomery-Åsberg Depression Rating Scale (MADRS).

## Methods

### Data Sources

Three trials that used objective cognitive tests to examine the effects of vortioxetine 5–20mg on cognitive dysfunction in MDD were included in the meta-analysis (Katona et al., 2012; McIntyre et al., 2014; Mahableshwarkar et al., 2015). These were similarly designed, 8-week, randomized, double-blind, placebo-controlled trials in patients who met the *Diagnostic and Statistical Manual of Mental Disorders* criteria for recurrent MDD (Fourth Edition, Text Revision; DSM-IV-TR; American Psychiatric Association, 2000b) and had a MADRS (Montgomery and Åsberg, 1979) total score ≥26 at screening and baseline visits. In all three trials, change in cognitive function was assessed using the DSST score, which integrates the function of a variety of cognitive domains (described above; Table 1). In two of the source studies (Katona et al., 2012; Mahableshwarkar et al., 2015), duloxetine 60mg/day was used as an active reference for assay sensitivity.

**Table 1.** Study Designs and Demographic Information

**Trial**	**Duration (weeks**)	**Key inclusion criteria**	**Randomized treatment groups**	**Primary endpoint**	**Key baseline demographics**
Mahableshwarkar et al., 2015	8	• Aged 18–65 years • Recurrent MDD according to DSM IV-TR criteria • Current MDE ≥3 months’ duration • MADRS total score ≥26 • Subjective report of cognitive dysfunction (e.g. difficulty with concentrating, thinking, learning/remembering new things) at baseline	• Vortioxetine 10– 20mg/day* (n = 198) • Duloxetine 60mg/day (n = 210) • Placebo (n = 194)	Change from baseline in DSST performance score	• 66.0% female • Mean age: 45.3 years • Median length of current MDE: 156 days • Mean MADRS score: 31.6
McIntyre et al., 2014	8	• Aged 18–65 years • Recurrent MDD according to DSM IV-TR criteria • Current MDE ≥3 months’ duration • MADRS total score ≥26	• Vortioxetine 10mg/ day (n = 195) • Vortioxetine 20mg/ day (n = 207) • Placebo (n = 196)	Composite cognition score comprising DSST^†^ and RAVLT	• 65.8% female • Mean age: 45.7 years • Median length of current MDE: 133 days • Mean MADRS score: 31.6
Katona et al., 2012	8	• Aged ≥65 years • Recurrent MDD according to DSM IV-TR criteria • Current MDE ≥4 weeks’ duration • MADRS total score ≥26	• Vortioxetine 5mg/day (n = 156) • Duloxetine 60mg/day (n = 151) • Placebo (n = 145)	Change from baseline in HAM-D_24_ total score (Cognitive effects were measured using RAVLT and DSST)	• 65.5% female • Mean age: 70.6 years • Median length of current MDE: 154 days • Mean MADRS score: 30.5

*Patients assigned to vortioxetine received 10mg/day on days 1–7 of the double-blind treatment period, with the option to increase to 20mg/day at the end of week 1 based on investigator judgment. For the remaining 7 weeks, the dose of vortioxetine was flexible at 10 or 20mg/day based on investigator judgment.

^†^DSST was a key secondary endpoint (multiplicity controlled) and was therefore subject to significance testing.

DSM-IV-TR, Diagnostic and Statistical Manual of Mental Disorders, Fourth Edition, Text Revision; DSST, Digit Symbol Substitution Test; HAM-D_24_, 24-item Hamilton Depression Scale; MADRS, Montgomery-Åsberg Depression Rating Scale; MDD, major depressive disorder; MDE, major depressive episode; RAVLT, Rey Auditory Verbal Learning Test.

### Outcomes and Assessments

In this meta-analysis, change in DSST score from baseline to 8 weeks was selected as the primary outcome measure. Improvement in certain depressive symptoms (e.g. energy, concentration, and focus) following antidepressant therapy indirectly improves cognitive function and can confound evaluation of the direct effects of the drug on cognitive function. To control for this possibility, change in the MADRS total score—reported in all three source studies—was analyzed separately and taken into account in the analyses of cognitive function.

At least one additional objective measure of cognitive function was assessed at 8 weeks in two of the three source trials, using either “pencil-and-paper” or computerized cognitive tests. Pencil-and-paper tests were the Trail Making Tests A and B (TMT-A and TMT-B), which assess processing speed and executive function, respectively; the Stroop Color Naming Test with congruent and incongruent stimuli (attention); and the acquisition and delayed recall subtests of the RAVLT (learning and memory). Several computerized tests were also included: Simple Reaction Time (SRT; to assess processing speed), Choice Reaction Time (CRT; attention), the Groton Maze Learning Test (GMLT; visual learning and memory), and the One-Back Test (OBT; attention and working memory). Although these outcomes were not included in the meta-analysis, data from the individual studies are presented.

### Data Analysis

Data were analyzed for a modified intent-to-treat set: the full analysis set (FAS) comprises all patients who received at least one dose of study medication and had at least one valid post-baseline assessment of the primary outcome. Change from baseline on DSST in patients treated with vortioxetine or duloxetine, each versus placebo, was analyzed using analysis of covariance (ANCOVA) using last observation carried forward (LOCF) to impute missing data. To assess the effects of vortioxetine (all doses combined, to account for the flexible [10/20 mg] dosing regimen used in Mahableshwarkar et al., 2015) and duloxetine on cognition, standard random effects meta-analysis was applied using all three trials, and separately using only the two duloxetine-referenced trials. To allow comparison of the magnitude of effects across different cognitive tests, and of different versions of the same test in different source studies, standardized effect sizes (SES) for vortioxetine/duloxetine versus placebo were used as input based on Cohen’s *d,* calculated as the mean difference from placebo divided by the standard deviation (SD) of the mean difference; the standard 0.2 threshold was used to determine clinical relevance (Cohen, 1988; Florea et al., 2015). Heterogeneity in source data was quantified using the I-squared (I^2^) test.

Path analysis (an extension of the multiple regression method used to describe the directed dependencies among a set of variables) was used to determine the extent to which cognitive score changes, as measured by DSST performance, were or were not mediated by change in MADRS scores (Ditlevsen, 2005a and 2005b). This was done by including the change from baseline in MADRS score in the ANCOVA model. The meta-analysis was applied to both MADRS-adjusted and -unadjusted SES values to allow interpretation of the independent (not mediated via improvement in mood symptoms, as assessed by MADRS) and dependent (mediated via improved mood) effects of vortioxetine and duloxetine on cognition, respectively.

Change in cognitive tests other than DSST at 8 weeks was analyzed using ANCOVA (FAS, LOCF) and the results are presented as SES with the 95% confidence interval (95% CI).

### Assessment of Bias

The likelihood that the true effect of each intervention was under- or over-estimated (i.e. risk of bias) was assessed for all three clinical trials included in the meta-analysis, based on recommendations from the Cochrane Handbook for Systematic Review of Interventions (www.cochrane-handbook.org). Areas of potential bias within five domains were examined: selection (i.e. presence of a rule for random allocation of interventions; strict implementation of random assignment by preventing foreknowledge of allocations); performance (i.e. blinding of study participants and personnel); detection (i.e. blinding of outcome assessors); attrition (i.e. intention-to-treat analysis being the least biased method of estimating the effects of an intervention); and other (e.g. for-profit bias). Risk of bias was considered high if, based on the protocol, bias in a given domain could not be excluded, or if there was no description of the domain within the primary publication. Trials for which an adequate protocol was described for a given domain were labeled low risk. There was no opportunity for publication bias since the three source studies were the first and only trials of cognitive function with vortioxetine at the time of this analysis.

### Ethical Statement

The study protocols and all related forms and amendments for each of the trials included in this meta-analysis were approved by the local independent ethics committee at each study center. All studies were conducted in accordance with the International Conference on Harmonization Good Clinical Practices guidelines, and with the ethical principles of the Declaration of Helsinki. All studies were also registered at ClinicalTrials.gov (Katona et al., 2012: NCT00811252; McIntyre et al., 2014: NCT01422213; Mahableshwarkar et al., 2015: NCT01564862).

## Results

### Study Characteristics

Baseline demographics for the 1657 randomized patients with recurrent MDD are shown in Table 1. The majority of patients were female, with a mean age of approximately 45 years in two trials (McIntyre et al., 2014; Mahableshwarkar et al., 2015) and 70 years in the third (Katona et al., 2012). Risk of bias was low across most domains; factors contributing to high risk were primarily in the for-profit domain, due to industry funding of all three trials, and the attrition domain in one trial (Mahableshwarkar et al., 2015).

### Effects of Vortioxetine on Cognitive Function: Data Unadjusted for Change in MADRS Score

The DSST score for vortioxetine-treated patients separated from placebo at all doses (5, 10, 20, and 10/25mg) in each of three individual trials, with SES ranging from 0.25 to 0.48 and a nominal *p*-value < 0.05 (Table 2). Vortioxetine also improved nearly all other measures of cognitive function that were tested, relative to placebo, in two of the three trials (RAVLT acquisition and RAVLT delayed recall in Katona et al., 2012, and McIntyre et al., 2014; TMT-A, TMT-B, Stroop incongruent, SRT, and CRT only in McIntyre et al., 2014; Table 3). In the third trial (Mahableshwarkar et al., 2015), improvement was seen in TMT-A, TMT-B, Stroop incongruent (RAVLT was not tested), and all the computerized tests, although not always to a statistically significant degree (Table 3).

**Table 2.** Change from Baseline in DSST After 8 Weeks (SES 593 Versus Placebo)

**Cognitive test**	Mahableshwarkar et al., 2015		McIntyre et al., 2014	Katona et al., 2012		**Meta-analysis (three studies**)	**Meta-analysis** **(two duloxetine-referenced studies)**	
	**Vortioxetine** **10/20 mg** **(n = 175)**	**Duloxetine** **60 mg** **(n = 187)**	**Vortioxetine** **10/20 mg** **(n = 397)**	**Vortioxetine** **5 mg** **(n = 152)**	**Duloxetine** **60 mg** **(n = 144)**	**Vortioxetine** **(n = 724)**	**Vortioxetine** **(n = 327)**	**Duloxetine** **(n = 331)**
***Data not adjusted for change in MADRS total mean score (assessment of both direct and indirect effects***)								
Difference vs placebo, SES ± SE [95% CI]	0.25±0.11 [0.04, 0.47]	0.18±0.11 [–0.03, 0.38]	0.48±0.09 [0.31, 0.65]	0.27±0.12 [0.04, 0.49]	0.07±0.12 [–0.16, 0.30]	**0.35±0.08** **[0.19, 0.50]**	**0.26±0.08** **[0.10, 0.41]**	**0.13±0.08** **[–0.03, 0.28]**
*p*-value	0.02	0.10	<0.0001	0.02	0.53	**<0.0001**	**0.001**	**0.10**
***Data adjusted for change in MADRS total score (assessment of direct effects only***)								
Difference vs placebo, SES ± SE [95% CI]	0.19±0.11 [–0.02, 0.41]	0.09±0.11 [–0.12, 0.29]	0.29±0.09 [0.12, 0.46]	0.20±0.12 [–0.03, 0.42]	–0.02±0.12 [–0.25, 0.21]	**0.24±0.06** **[0.12, 0.35]**	**0.19±0.08** **[0.04, 0.35]**	**0.04±0.08** **[–0.12, 0.19]**
*p*-value	0.07	0.42	0.001	0.09	0.88	**<0.0001**	**0.01**	**0.62**

CI, confidence interval; DSST, Digit Symbol Substitution Test; MADRS, Montgomery-Åsberg Depression Rating Scale; SE, standard error; SES, standardized effect size.

Mahableshwarkar et al. (2015) and McIntyre et al. (2014) included change in DSST as primary or multiplicity-controlled secondary endpoints, whereas in Katona et al. (2012) change in DSST was a secondary endpoint (not multiplicity-controlled). DSST *p* -values from the Katona et al. (2012) trial are therefore nominal.

**Table 3.** Change from Baseline in Various Additional Cognitive Tests After 8 Weeks of Vortioxetine or Duloxetine Treatment (Standardized Effect Size Versus Placebo)

**Cognitive test**	Mahableshwarkar et al., 2015		McIntyre et al., 2014		Katona et al., 2012	
	**Vortioxetine** **10/20 mg** **(n = 175)**	**Duloxetine** **60 mg** **(n = 187)**	**Vortioxetine** **10 mg** **(n = 193)**	**Vortioxetine** **20 mg** **(n = 204)**	**Vortioxetine** **5 mg** **(n = 152)**	**Duloxetine** **60 mg** **(n = 144)**
**Paper-and-pencil tests**						
TMT-A	0.08	0.11	0.28**	0.29**	–	–
TMT-B	0.38*	0.22	0.28**	0.31**	–	–
STROOP congruent	–0.08	0.01	0.32**	0.33**	–	–
STROOP incongruent	0	0.09	0.33**	0.31**	–	–
RAVLT acquisition	–	–	0.26*^†^	0.14^†^	0.27*	0.33**
RAVLT delayed recall	–	–	0.32**	0.28**	0.24*	0.32**
**Computerized tests**						
SRT	0.16	0.06	0.42***	0.24*	–	–
CRT	0.18	0.09	0.36***	0.10	–	–
GMLT	0.11	0.10	–	–	–	–
OBT	0.08	0.04	–	–	–	–

*p < 0.05; **p < 0.01; ***p < 0.001 vs placebo. All *p*-values are nominal unless stated otherwise. ^†^ *p*-value not nominal.

CRT, Choice Reaction Time; DSST, Digital Symbol Substitution Test; GMLT, Groton Maze Learning Test; OBT, One-back Test; RAVLT, Rey Auditory Verbal Learning Test; SRT, Simple Reaction Time; STROOP, Stroop Color Naming Test; TMT-A/B, Trail Making Tests A and B.

**Figure 1.**
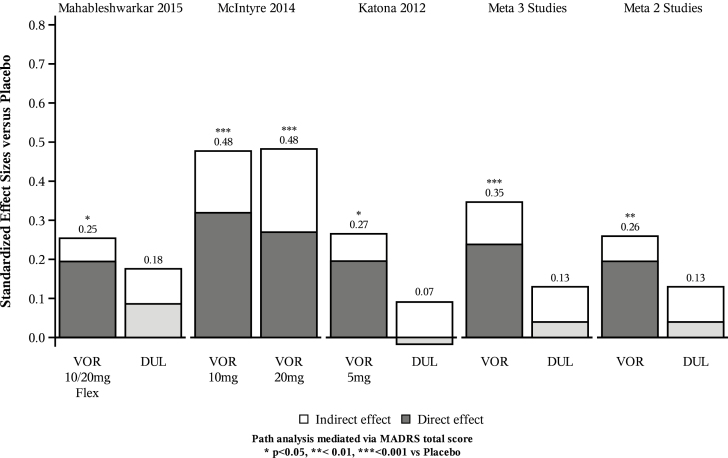
Total, direct, and indirect effects of vortioxetine and duloxetine on Digital Symbol Substitution Test adjusted for change from baseline in MADRS score (path analysis). *p < 0.05, **p < 0.01, ***p < 0.001 vs placebo. Numbers above bars are standardized total effect size versus placebo. *P*-values refer to results from unmediated ANCOVA model that did not include change in MADRS. Meta 3 studies: meta-analysis based on Mahableshwarkar et al. (2015), McIntyre et al. (2014), and Katona et al. (2012). Meta 2 studies: meta-analysis based on Mahableshwarkar et al. (2015) and Katona et al. (2012). Indirect effect: correlated with MADRS; direct effect: not correlated with MADRS. ANCOVA, analysis of covariance; DUL, duloxetine; MADRS, Montgomery-Åsberg Depression Rating Scale; VOR, vortioxetine.

In the meta-analysis of all three trials, vortioxetine significantly improved performance on the DSST compared with placebo (SES 0.35; 95% CI: 0.19, 0.50; *p* < 0.0001; Table 2; Figure 1). Similarly, in the meta-analysis of the two duloxetine-referenced trials, separation from placebo on DSST performance was statistically significant for vortioxetine (SES 0.26; 95% CI: 0.10, 0.41; *p* = 0.001). Conversely, duloxetine did not lead to a statistically significant change in DSST relative to placebo in either of the individual trials or in the meta-analysis of both (SES 0.13; 95% CI: –0.03, 0.28; *p* = 0.10; Table 2). The I^2^ value in the meta-analysis of unadjusted data for the three trials was 42% (data not shown), indicating a low likelihood that study heterogeneity had a relevant impact on effect estimates (Higgins et al., 2003). In the meta-analysis of the two duloxetine-referenced trials, the I^2^ value of 0% indicated no heterogeneity (data not shown).

### Effects of Vortioxetine on Cognitive Function: Data Adjusted for Change in MADRS Score

Mean reduction in depressive symptoms, assessed by the MADRS total mean score at 8 weeks (vortioxetine: 14.8–17.6 points; duloxetine: 15.8–19.2 points), was significantly greater with both active treatments versus placebo in all three trials (vortioxetine difference versus placebo, 2.3–6.7 points; duloxetine difference versus placebo: 3.3–7.6; all *p* < 0.05). After adjustment for change in the MADRS total mean score, improvement in DSST remained significantly greater with vortioxetine relative to placebo in one trial (McIntyre et al., 2014) and there was a similar, but non-significant, trend in the other two trials (Table 2). As expected, in duloxetine-treated patients, improvement in the DSST score was not significant after adjustment for change from baseline in the MADRS total score in either trial in which it was an active reference (Table 2).

**Figure 2.**
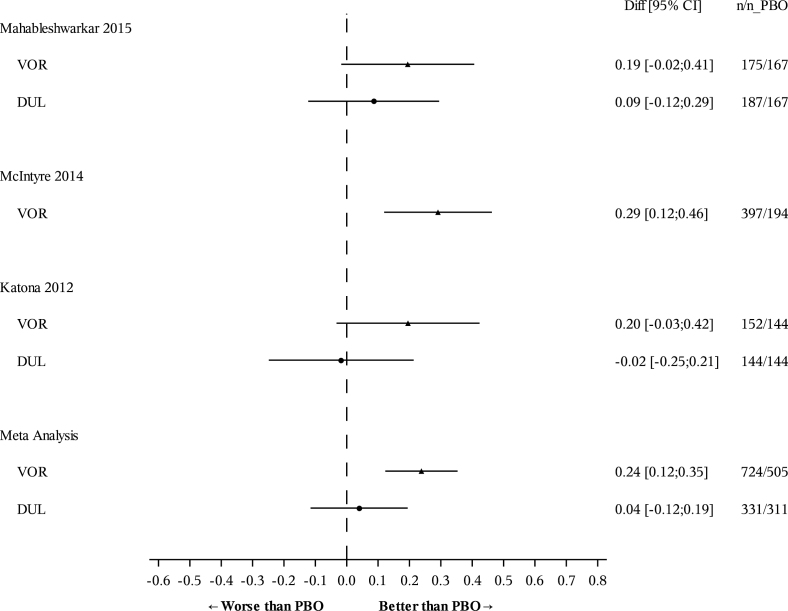
Change in DSST (number of correct symbols; SES versus placebo) from baseline to 8 weeks after adjustment for change in MADRS total score. Data are full analysis set (FAS), ANCOVA, last observation carried forward (LOCF). ANCOVA, analysis of covariance; CI, confidence interval; DSST, Digital Symbol Substitution Test; DUL, duloxetine; MADRS, Montgomery-Åsberg Depression Rating Scale; PBO, placebo; SES, standardized effect size; VOR, vortioxetine.

In the meta-analysis of three trials, separation from placebo in the DSST score remained consistent in vortioxetine-treated patients after the DSST score was adjusted for change in the MADRS score, with an SES of 0.24 (95% CI: 0.12, 0.35; *p* < 0.0001; Table 2; Figure 2). Similarly, in the meta-analysis of the two duloxetine-referenced trials, vortioxetine maintained a significant difference from placebo after adjustment for change in MADRS score (SES 0.19; 95% CI: 0.04, 0.35; *p* = 0.01; Table 2). By contrast, there was no separation from placebo in the duloxetine group (SES 0.04; 95% CI: –0.12, 0.19; *p* = 0.62; Figure 2). Comparison of vortioxetine versus duloxetine revealed a statistically significant difference in DSST scores in favor of vortioxetine after adjustment for change in MADRS score (SES for vortioxetine relative to duloxetine = 0.16; 95% CI: 0.004, 0.310; *p* = 0.04). The I^2^ value in both meta-analyses was 0%, indicating no heterogeneity (data not shown).

## Discussion

In this meta-analysis of three similarly designed, well-powered, randomized, double-blind, placebo-controlled, 8-week trials, vortioxetine 5–20mg/day was associated with significant improvements in cognitive function compared with placebo, as measured by performance on DSST (Figure 1). These effects were independent of the beneficial effects of vortioxetine on depressive symptoms, with separation from placebo maintained after adjustment for change from baseline in the MADRS total score. Similarly, meta-analysis of the two studies that included both vortioxetine and duloxetine indicated statistically significant improvements in DSST performance relative to placebo for vortioxetine but not duloxetine, both before and after adjustment for change in depressive symptoms. Moreover, there was a statistically significant difference in DSST scores in favor of vortioxetine compared with duloxetine, even after adjustment for change from baseline in the MADRS total score. The present analysis is the first with sufficient statistical power and methodological rigor to provide robust evidence of the independent effects of vortioxetine, or indeed any antidepressant, on cognitive function in MDD. Showing that the favorable effects of vortioxetine on cognitive function were independent of antidepressant effects gives further support to the results of the individual trials.

Assessing the key outcome, the consistency and magnitude of the standardized effect size of vortioxetine relative to placebo on cognitive test outcomes in the individual trials (SES range 0.25–0.48, Cohen’s *d*; Table 2) and on meta-analysis (SES 0.35; Figure 1), supports the fact that vortioxetine can improve cognitive function with a small to moderate degree of clinical significance. To put these values in context, the SES of treatment with vortioxetine were of similar magnitude to those of the cognitive deficits (measured using DSST and other tests) observed in patients with MDD relative to healthy controls, for which meta-analyses indicate that the typical SES are 0.2–0.7 (Lee et al., 2012; Snyder, 2013; Rock et al., 2014), a magnitude of cognitive decline that is anticipated to have a profound impact on occupational and school performance. Moreover, the observed SES with vortioxetine are consistent with, and similar to, those reported for many drugs used in general medical and psychiatric practice (e.g. paroxetine, fluoxetine, and lithium; Leucht et al., 2012), and in disorders of cognition (e.g. memantine in Alzheimer’s Disease; SES = 0.27; Matsunaga et al., 2015). They are also in accordance with effect sizes for recognized non-pharmacological cognitive interventions such as cognitive remediation therapy in schizophrenia (SES = 0.34; Wykes et al., 2007).

Clinical relevance can also be gauged by comparison with SES for psychoactive agents for which there are label warnings or government-mandated limits due to their potential impact on public safety; examples include alcohol and sedating drugs in people operating large vehicles or dangerous machinery. Studies of DSST performance relative to placebo have shown effect sizes (Cohen’s *d*) of 0.25–0.5 for diphenhydramine (Roth et al., 1987) and lorazepam (Pompeia et al., 2008), and 0.68 in people whose blood alcohol concentration is 0.09% (Mattila and Mattila-Evenden, 1997; national guidelines usually stipulate an upper threshold of either 0.05 or 0.08%; http://www.drinkdriving.org/worldwide_drink_driving_limits.php). Importantly, while the effects of these agents cease once the drug effects wear off, the effects of cognitive dysfunction in chronic MDD are far more persistent.

The present meta-analysis has some important strengths and limitations. Strengths include the similar design of the three source studies, the large number of patients included, and the consistency of effect of vortioxetine on cognitive dysfunction in the individual trials and meta-analyses. Although test outcomes and SES in clinical trials may be influenced by demographic and methodological factors such as the composition of the trial population, variation in testing procedures, and regional differences in populations and clinical practice norms, formal testing for heterogeneity in the current dataset supported the validity of the results of this meta-analysis. Use of path analysis makes this the first meta-analysis to distinguish between the independent and mood-associated effects of two antidepressants on cognitive function. In addition, risk of bias was assessed and was low across most domains, with the exception of industry funding, which was disclosed in all cases.

Study limitations include the single cognitive outcome measure (DSST) and the fact that functional outcomes were not consistently examined. These facts notwithstanding, vortioxetine improved other measures of cognitive performance in the individual trials, lending support to the conclusion. Furthermore, one of the source trials (Mahableshwarkar et al., 2015) used the objective University of California San Diego Performance-based Skills Assessment (UPSA; Patterson et al., 2001) to measure functional capacity across five domains (household chores, communication, finance, transportation, and planning recreational activities), and the patient-reported Working Limitation Questionnaire (WLQ) to measure workplace performance across five domains (productivity loss, time management, and physical, mental, and output demands). In the Mahableshwarkar et al. trial (2015), vortioxetine, but not duloxetine, demonstrated significant improvements in UPSA composite functional scores (UPSA–Brief in EU patients and UPSA–Validation of Intermediate Measures in US patients) and cognitive test scores, relative to placebo. Although the study was not powered to detect differences in workplace outcomes between vortioxetine or duloxetine and placebo in the subset of working patients, both active agents showed numerical advantage over placebo on multiple WLQ subscales and reduced productivity losses. Notably, vortioxetine was statistically significantly superior to placebo in decreasing the difficulty of time management, which is closely correlated with cognitive function (Ward et al., 2012; Mioni et al., 2013).

Further work is necessary to characterize in detail the cognitive effects of vortioxetine and identify which patient subgroups (by age, gender, genotype, etc.) can benefit most from these effects. In addition, longer term studies will be needed to better understand the impact of treating cognitive dysfunction on long-term outcomes in MDD, such as functional recovery or development of dementia or other cognitive disorders. Additional investigations will also be necessary to elucidate the molecular mechanisms underlying the cognitive effects of vortioxetine in MDD, which are currently only partially understood but may stem from its unique multimodal mechanism of action. Studies in animal models have suggested that increased firing of pyramidal neurons in the medial prefrontal cortex and enhanced hippocampal long-term potentiation may be involved (Haddjeri et al., 2012; Riga et al., 2013; Pehrson et al., 2015). Studies are ongoing to provide further understanding of the effects of vortioxetine on cognitive dysfunction in MDD.

In conclusion, in this meta-analysis of three randomized controlled trials in patients with MDD, vortioxetine significantly and consistently improved cognitive function, as assessed by DSST scores, before and after adjustment for change in depressive symptoms. The improvements in cognitive test outcomes observed with vortioxetine were not apparent with duloxetine. These results were supported by a range of additional tests of various cognitive domains, strengthening the conclusion that vortioxetine has a direct effect on cognitive performance in patients with MDD, independent from its antidepressant effects. Vortioxetine may therefore represent an important treatment option in patients with cognitive dysfunction associated with MDD.

## Statement of Interest

Dr McIntyre is a consultant to and receives speaker fees from Takeda, Lundbeck, AstraZeneca, Eli-Lilly, Janssen, Otsuka, Sunovion, Allergan, and Pfizer. In the past two years, Dr Harrison has received honoraria and paid consultancy from Abbvie, A2Q, Amgen, Anavex, AstraZeneca, Avraham, Axon, Axovant, Biogen Idec, Boehringer Ingelheim, Bracket, Catenion, CRF Health, DeNDRoN, EnVivo Pharma, Enzymotec, ePharmaSolutions, Eisai, Eli Lilly, Forum Pharma, GfHEu, Heptares, Janssen AI, Johnson & Johnson, Kaasa Health, Kyowa Hakko Kirin, Lundbeck, MedAvante, Merck, MyCognition, Mind Agilis, Neurocog, Neurim, Neuroscios, Neurotrack, Novartis, Nutricia, Orion Pharma, Pharmanet/i3, Pfizer, Prana Biotech, PriceSpective, Probiodrug, Prophase, Prostrakan, Regeneron, Reviva, Roche, Sanofi, Servier, Shire, Takeda, TCG, TransTech Pharma, and Velacor. Drs Loft and Olsen are employees of H Lundbeck A/S. Dr Jacobson is an employee of Takeda Pharmaceutical Company, Ltd.
